# Preclinical testing of antimicrobials for cystic fibrosis lung infections: current needs and future priorities

**DOI:** 10.1099/mic.0.001361

**Published:** 2023-07-10

**Authors:** Lucile Hubert, Thomas E. Barton, Hollie J. Leighton, Brogan Richards

**Affiliations:** ^1^​ Microbiomes, Microbes and Informatics Group, Organisms and Environment Division, Cardiff School of Biosciences, Cardiff University, Sir Martin Evans Building, Park Place, Cardiff, UK; ^2^​ Department of Clinical Infection, Microbiology and Immunology, University of Liverpool, Ronald Ross Building, 8 West Derby Street, Liverpool, L69 7BE, UK; ^3^​ School of Life Sciences, University of Nottingham, Nottingham, UK

**Keywords:** CF community, cystic fibrosis, model development

## Abstract

A workshop was held by the PIPE-CF strategic research centre to consider preclinical testing of antimicrobials for cystic fibrosis (CF). The workshop brought together groups of people from the CF community to discuss current challenges and identify priorities when developing CF therapeutics. This paper summarizes the key points from the workshop from the different sessions, including talks given by presenters on the day and round table discussions. Currently, it is felt that there is a large disconnect throughout the community, with communication between patients, clinicians and researchers being the main issue. This leads to little consideration being given to factors such as treatment regimes, routes of administration and side effects when developing new therapies, that could alter the day-to-day lifestyles of people living with CF. Translation of numerical data that are obtained in the laboratory to successful outcomes of clinical trials is also a key challenge facing researchers today. Laboratory assays in preclinical testing involve basing results on bacterial clearance and decrease in viable cells, when these are not factors that are considered when determining the success of a treatment in the clinic. However, there are several models currently in development that seek to tackle some of these issues, such as the organ-on-a-chip technology and adaptation of a hollow-fibre model, as well as the development of media that aim to mimic the niche environments of a CF respiratory tract. It is hoped that by summarizing these opinions and discussing current research, the communication gap between groups can begin to close.

## Introduction

On 20 October 2022, the PIPE-CF strategic research centre, in collaboration with the UK Cystic Fibrosis infection biorepository (UKCFIB, https://cfamr.org.uk/), held a workshop to consider preclinical testing of antimicrobials for cystic fibrosis (CF) respiratory tract infections. The workshop brought together individuals from industry and academia, clinicians and people with CF (PwCF) to discuss the current challenges in preclinical antimicrobial development, and identify priorities in future preclinical development of CF antimicrobials. The workshop was designed as an agenda-setting exercise, focusing on the need for innovation and optimization of preclinical models, to enhance the CF antimicrobial medicine discovery pipeline.

## Aims and expected outcomes

The workshop facilitated collaboration between researchers involved in PIPE-CF and UKCFIB to enable greater constructive collaboration in future research. Early career researchers took a leading role in the workshop, gaining a broad perspective on antimicrobial development in the CF space, and helping them better define their own research agenda in CF and antimicrobial research. The workshop aimed to identify future outputs and support that were needed by the community, thereby helping to refine the PIPE-CF and UKCFIB research programmes.

## Perspectives – key considerations for preclinical development

Chronic and acute infections pose a considerable burden to PwCF, with 95 % of CF-related morbidity and mortality being associated with progressive lung disease and respiratory failure [1]. While antibiotics are available, the resolution of chronic infections is currently unfeasible, despite PwCF following complex regimes of antimicrobial therapy [1]. Delegates highlighted a lack of new antimicrobial therapeutics being developed that target CF lung infections and emphasized that many new agents that are efficacious in preclinical development show limited clinical benefits.

A key theme from the meeting was the necessity for researchers to understand the perspectives of the CF community. The characteristics of new antibiotics, including the route of administration and potential side effects, must be taken into consideration to avoid an unnecessary increase in the treatment burden for PwCF. Side effects include gastrointestinal reactions, skin reactions (dermatitis, itching, rash and others), nephrotoxicity, etc. [2]. Collaborations with the CF community exist, such as the James Lind Alliance (JLA), which through its Priority Setting Partnerships has worked with PwCF, their families and healthcare professionals to define specific priorities in CF research [3]. The JLA top 10 research priorities were defined in 2017, and recently refreshed, with a focus on managing an ageing population and identifying long-term and systemic impacts of modulators. The CF Trust also has a considerable focus on outreach, organizing focus groups, conducting review groups for proposed trials and providing a trial tracker, allowing PwCF to identify clinical trials that may suit themselves or those around them [4]. Researchers must continue to interface with the community so as to answer previously overlooked questions, those pertaining to the psychological impact of therapeutics, the factors most important to quality of life (QoL), and how treatment management may be optimized.

When developing CF antimicrobials, translation from preclinical to clinical outcomes is paramount. Clinical metrics for drug efficacy are limited to QoL assessments and the maximum forced expiratory volume in one second (FEV_1_) as a measure of lung function. These cannot be assessed in preclinical testing, where bacterial clearance is relied upon. Based on this metric, drugs are erroneously selected for further testing, despite clearance rarely being achieved in the clinic. Rather than focusing on resolution of infection, antimicrobial development could focus on resolution of pulmonary exacerbation, to limit the impact of exacerbations of FEV_1_ and QoL. With this focus, antimicrobial efficacy should be evaluated by assessing changes in host and pathogen biomarkers associated with clinical resolution of pulmonary exacerbation. Preclinical models mimic different factors in *in vitro* host–pathogen systems and *in vivo* models [5]. These models should be optimized to incorporate relevant CF-specific components, while being assessed and optimized for their accuracy in expressing biomarkers predictive of clinical success. This will provide a suite of preclinical models that accurately predict the impact of potential antimicrobials in resolving pulmonary exacerbations.

In formulating new antibiotics, delegates emphasized the need to consider the mode of action and long-term safety of new drugs and their impact on the population dynamics and host–pathogen interactions in the CF lung microbiome. Inhaled antimicrobials offer a mode of action that is less invasive than parental antibiotics, while reducing hypersensitivity, which the latter causes in 20–60 % of PwCF [6, 7]. Inhaled colistin and tobramycin are the most prescribed CF antibiotics in the UK for *Pseudomonas aeruginosa* infection, although hypersensitivity still occurs, and penetration into the lungs is limited [2]. The development of antivirulence drugs and phage therapy and the repurposing of existing drugs may also prove efficacious. In combination, these alternative therapeutics have the potential to improve clinical outcomes for PwCF.

## Research – identifying the right clinical isolates

CF lung infections are polymicrobial, comprising different species of bacteria, fungi and viruses. The most common CF pathogens are well-known and characterized and include Gram-negative and Gram-positive multidrug-resistant species. These are *P. aeruginosa *, *Burkholderia cepacia* complex (Bcc) and *Burkholderia gladioli *, *Staphylococcus aureus *, *Mycobacterium abscessus* complex, *Achromobacter* and others [8]. However, knowledge gaps persist for other organisms, primarily emerging fungal pathogens, such as *Scedosporium* sp. or *Lomentospora* sp. and anaerobes, including *Prevotella* sp. and *Veillonella* sp. [9]. Curating a species and strain panel of CF pathogens for antimicrobial development requires consideration of their prevalence worldwide. This has become relevant for *Burkholderia* species in recent years, with the increasing prevalence of *B. gladioli* observed mostly in the USA [10].

Obtaining genomic and phenotypic information on species of interest is crucial to avoid selecting strains that are irrelevant to the research. Extensive genomic analyses of both clinical and environmental isolates of *P. aeruginosa* have revealed the existence of five genomic groups, with the majority belonging to groups 1 and 2 [11]. Group 3 isolates included the multidrug-resistant PA7 strain and possessed an average nucleotide identity (ANI) below the 95 % boundary, indicating that this group could be considered as a novel genomic species and thus not representative of CF strains [11, 12]. Similar work was conducted on *Burkholderia cenocepacia* and what was formerly known as *B. cenocepacia* subgroup B now possesses its own genomic species called *Burkholderia orbicola* [13], whilst the proposed name of *Burkholderia servocepacia* for subgroup A has not been an accepted taxonomic change [14]. Similarly, pheno-genomic studies of *M. abscessus* have enabled the identification of dominant clones that are able to transmit between CF and non-CF populations [15]. Genome sequencing has revealed the existence of consensus single-nucleotide polymorphisms (SNPs) in *M. abscessus* isolates that are responsible for the acquisition of novel phenotypes, characteristic of chronic infections [16].

The existence of large repositories, such as the *Burkholderia cepacia* Research Laboratory and Repository led by Professor John LiPuma, regrouping over 55 000 *Burkholderia* isolates from the environment, CF lung infections and non-CF infections, and the repository led by Professor Jane Davies comprising 11 000 isolates collected from CF clinical samples, enables researchers to work with many strains to investigate spatial and temporal changes or specific phenotypes, including biofilm, virulence factor production, antibiotic susceptibility, etc. This allows a better understanding of how different strains would respond to antimicrobials and their potential mechanisms of resistance.

## Research – developing and using preclinical models that predict clinical success

New therapeutics are rarely tested under conditions that mimic the conditions pathogens are exposed to in =CF lungs, making it difficult to predict clinical success for new CF antimicrobials. Various methods to create CF-mimicking models were introduced during the final session as potential systems for preclinical trials.

Organ-on-a-chip technology [17] aims to model human organs using microfluidic channels, separated by membranes with different levels of permeability to allow communication between cell types. CF-like conditions can be reproduced with the addition of an extracellular matrix and controlled oxygen conditions [18]. Miniaturized sensors on these chips allow real-time monitoring and rapid adjustments to pH, substrate levels, etc. [19]. The use of human cells in the chip can help detect misleading toxicological or pharmacological effects of the drugs tested, caused by species-specific characteristics. The organ-on-a-chip systems can also be differentiated to be representative of niche areas within a particular organ. For example, different groups have used the organ-on-a-chip to create a musculature model as a prerequisite for bronchoconstriction and bronchodilation [20], and another system has been used to reproduce the alveolar environments, which could be used to model the more chronic nature of cystic fibrosis [21].

Liquid culture media have been developed for the last couple of decades with the aim of reproducing the specific conditions of CF sputum and of the CF airways. These media, such as the artificial sputum medium (ASM) or the synthetic cystic fibrosis medium 2 (SCFM2), contain sugars, proteins, DNA, etc. at concentrations found in CF lungs [22–24]. SCFM2 was found to be able to reproduce similar gene expression levels to those identified in sputum samples for *P. aeruginosa *, including when compared to a murine lung infection model [25]. Recent media have been formulated to mimic conditions found in both the lungs and sinuses of pwCF [26]. These media contain elevated glucose concentrations to replicate CF-associated diabetes, and bile salts, which are a comorbidity of CF-associated gastrointestinal reflux disease. These biomimetic media were designed to better our understanding of the pressure bacterial communities experience when colonizing PwCF and the resulting adaptations.

Another challenge faced in preclinical development of drugs is discovering ways to research into the pharmacokinetics and pharmacodynamics in CF-relevant *in vivo* models. Acute models in mice do not necessarily take into consideration the chronic nature of CF lung infections. They ignore the importance of biofilms by focusing on (i) inoculations of the lung tissues with planktonic cultures and (ii) 24 h end points to determine drug efficiency, and so conducting research into the long-term effects on the drug and aspects such as the half-life and metabolism becomes difficult without progressing the drug in development to clinical trials. One proposed method to remedy these issues when studying pharmacokinetics and pharmacodynamics was to adapt the hollow fibre infection model, which is an *in vitro* closed system that allows bacteria to culture continuously, and precisely mimics human concentration–time profiles for the antimicrobial drug candidate [27, 28]. The model can be adapted to use biomimetic media and CF-specific isolates within the system.

## Conclusion

Numerous barriers to the development of novel antimicrobials exist, including a lack of relevant models and inconsistent preclinical results. The workshop aimed to facilitate collaboration between specialists associated with preclinical CF antimicrobial development, consolidate research priorities, and improve the efficiency and reproducibility of future developments.

The core themes/results that emerged from the workshop included the need to strengthen communication between PwCF, researchers and clinicians and develop a preclinical testing pipeline that more accurately represents the CF environment, as there is currently a lack of PwCF representation. Another key finding was the need to identify biomarkers of positive clinical outcomes, as the current methods do not reflect patient responses to the antimicrobials. In summary, standardization of the preclinical antimicrobial development pipeline is crucial to accelerate antimicrobial development and reduce the burden of infection in the CF community.
